# A Method for Fault Detection and Diagnostics in Ventilation Units Using Virtual Sensors †

**DOI:** 10.3390/s18113931

**Published:** 2018-11-14

**Authors:** Claudio Giovanni Mattera, Joseba Quevedo, Teresa Escobet, Hamid Reza Shaker, Muhyiddine Jradi

**Affiliations:** 1 Center for Energy Informatics (CFEI), Maersk Mc-Kinney Moller Institute (MMMI), University of Southern Denmark (SDU), 5230 Odense, Denmark; hrsh@mmmi.sdu.dk (H.R.S.); mjr@mmmi.sdu.dk (M.J.); 2 Center for Supervision, Security and Automatic Control (CS^2^AC), Polytechnic University of Catalonia (UPC), 08034 Barcelona, Spain; joseba.quevedo@upc.edu (J.Q.); teresa.escobet@upc.edu (T.E.)

**Keywords:** fault detection and diagnosis, virtual sensors, HVAC, smart buildings

## Abstract

Buildings represent a significant portion of global energy consumption. Ventilation units are complex components, often customized for the specific building, responsible for a large part of energy consumption. Their faults impact buildings’ energy efficiency and occupancy comfort. In order to ensure their correct operation, proper fault detection and diagnostics methods must be applied. Hardware redundancy, an effective approach to detect faults, leads to increased costs and space requirements. We propose exploiting physical relations inside ventilation units to create virtual sensors from other sensors’ readings, introducing redundancy in the system. We use two different measures to detect when a virtual sensor deviates from the physical one: coefficient of determination for linear models, and acceptable range. We tested our method on a real building at the University of Southern Denmark, developing three virtual sensors: temperature, airflow, and fan speed. We employed linear regression models, statistical models, and non-linear regression models. All models detected an anomalous strong oscillation in the temperature sensors. Readings fell outside the acceptable range and the coefficient of determination dropped. Our method showed promising results by introducing redundancy in the system, which can benefit several applications, such as fault detection and diagnostics and fault-tolerant control. Future work will be necessary to discover thresholds and set up automatic fault detection and diagnostics.

## 1. Introduction

In Europe, buildings account for 40% of the total energy used and 36% of the total CO_2_ emissions [1]. In the United States, the building sector accounted for about 41% of primary energy consumption in 2010, 44% more than the transportation sector and 36% more than the industrial sector. Total building primary energy consumption in 2009 was about 48% higher than consumption in 1980, going from 1290 TW h to 2784 TW h [2].

Modern buildings consist of different subsystems such as *heating, ventilation and air-conditioning* (HVAC) and lighting. Each subsystem contains, in turn, several components such as pumps, fans, ducts, sensors, lamps, wires etc. monitored and managed by a building management system. All these components are subject to faults, due to damage, wearing over time, misconfiguration, and communication issues. Faults impact occupancy, maintenance cost and particularly energy efficiency. It is estimated that in 2009 just 13 of the most common faults were responsible for over $3.3 billions in energy loss [3,4].

HVAC load varies depending on building type and location, but they are one of the critical subsystems and can make up to 50% of the total energy consumption and, therefore, faults involving them cause large energy loss [4,5]. Research suggests that between 20% to 30% energy saving could be achieved by re-commissioning malfunctioning HVAC systems [6]. HVAC systems are often customized for their specific building and, therefore, lack quality system integration [7].

Fault detection and diagnostics (FDD) techniques can be used to monitor building systems and to detect and diagnose anomalies and faults. FDD has been an active research area for many decades in fields such as process operations [8], avionics [9] or water distribution [10,11], and in the past few years has caught the interest in the field of buildings technology [12,13,14].

### Problem Statement

Building energy efficiency and safety cannot be achieved without FDD methods applied to ventilation units. Hardware redundancy is an effective approach to high-quality FDD; however, duplicating sensors and other components inside every unit increases deployment and maintenance costs, necessary space, and complexity. Commercial ventilation units are rarely shipped with hardware redundancy.

In this paper, we propose a mixed model-based and data-driven technique to exploit spatial relations among different components in ventilation units to create *virtual sensors* and introduce redundancy in the system, which can be used to detect and diagnose faults. For each considered sensor, we train a model to estimate its readings given other sensors in the unit. This allows us to detect and diagnose faults that cause physical and virtual sensors to deviate from each other. In addition to linear regression models, covered in previous work [15], in this paper we consider also autoregressive moving average with exogenous variables (ARMAX) models from statistical analysis and non-linear models such as support vector machine (SVM) regression and artificial neural network (ANN). We define two measures to detect when physical and virtual sensors deviate. We apply this technique to a real-world building and report the results.

The rest of the paper is organized as follows. The state of the art is reviewed in Section 2. The proposed technique is introduced in Section 3. Section 4 presents the case study and discusses results and implications. Finally, conclusions are drawn in Section 5.

## 2. State of the Art

### 2.1. Fault Detection and Diagnostics

**Figure 1:**
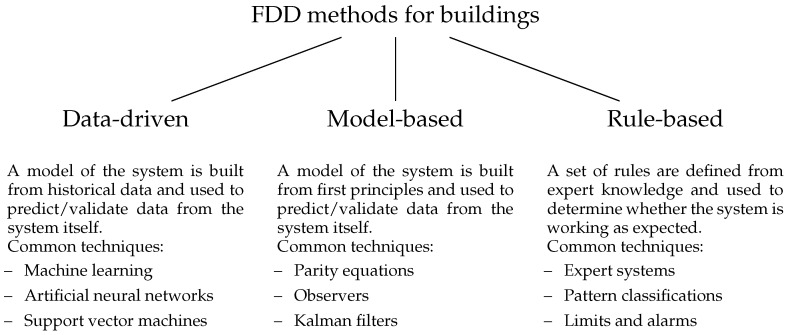
Categorization of FDD methods for buildings adapted from Kim and Katipamula [14].

Kim and Katipamula present a comprehensive review of recent FDD methods for building systems [14]. FDD methods are categorized into three groups depending on the approach: data-driven methods, model-based methods, and rule-based methods, as shown in Figure 1.

In data-driven methods, a model of the system under test is trained from historical data and it is used to validate current data from the system. Several techniques exist, such as machine learning, artificial neural network (ANN) or support vector machine (SVM). Little to no physical knowledge of the system is required and the resulting models can be treated as black-box components. For this reason, data-driven methods are easily applicable to several types of systems with small effort. However, historical data are necessary to create the model, which rules out the possibility to apply these techniques to newly deployed systems. These methods require fault-free training data, otherwise the generated models would recognize faults as correct behavior. To perform proper diagnostics and identify the precise fault, labelled faulty historical data is usually necessary.

In model-based methods, a physical model of the system under test is created from first principles and it is used to validate current data from the system. This approach does not require training data and often predictions are more accurate than black-box models. However, accurate models can be complex and require in-depth knowledge of the system and large effort to be created. Often it is necessary to perform parameters estimation to improve accuracy, which might require historical data and, therefore, prevent to use the model with newly deployed systems.

In rule-based methods, expert knowledge gathered from field experts is used to design a set of rules describing the system’s behavior. No historical data and no detailed physical knowledge of the system are necessary. Moreover, some faults have effects that can be described by rules, which makes it possible to precisely identify and diagnose the problem. However, rules can only describe behaviors up to a certain complexity and they can only cover simple cases. As the number of rules grows, the possibility of conflicting rules increases and so does the effort to maintain the set of rules.

**Figure 2:**
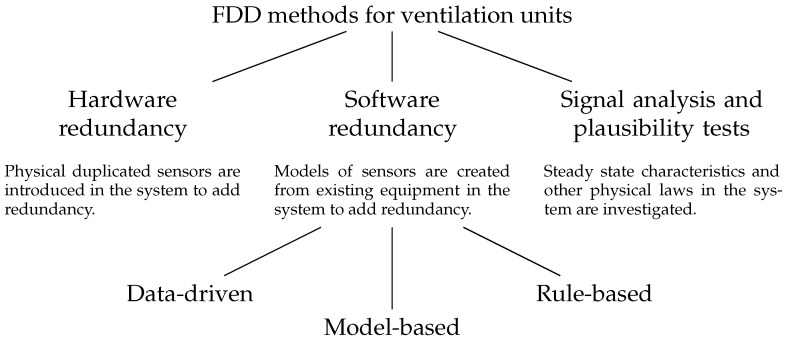
Classification of FDD techniques for ventilation units according to Yu et al. [7].

Yu et al. present a review of FDD techniques for ventilation units [7]. In this case, the authors classify FDD techniques into four groups: hardware redundancy, software redundancy, signal analysis and plausibility tests, as shown in Figure 2. Multiple identical sensors and actuators lead to hardware redundancy, which allows high accuracy and precision, but also to higher deployment and maintenance costs. In software redundancy, multiple physical sensors are replaced by models obtained by other sensors in the system. In signal analysis and plausibility tests methods, the steady-state characteristics and other physical laws in the system are investigated. Software redundancy methods are further classified in model-based, data-driven, and rule-based, as in general FDD methods.

The authors also define a list of desirable characteristics of FDD methods:

1. Quick detection and diagnostics: faults should be identified as soon as possible;
2. Isolability: the ability to distinguish between multiple faults, i.e., performing diagnostics;
3. Robustness: the method should be insensitive to noise and model uncertainties;
4. Novel identifiability: the ability to detect unknown faults;
5. Classification error estimate: the method should make its accuracy explicit, e.g., by having a confidence range as output;
6. Adaptability: the ability to automatically adapt to changes in the system under test;
7. Explanation facility: the ability to identify the precise location and cause of faults;
8. Modeling requirements: lower modeling requirements ease implementation and application on real-time processes;
9. Storage and computational requirements: minimal storage and computational requirements are necessary for an easy implementation and application on real-time processes;
10. Multiple fault identifiability: the ability to diagnose multiple simultaneous faults.

### 2.2. Virtual Sensors

Virtual sensors have been used successfully in various fields, both for observing hidden unmeasured quantities in the system and for validating the system’s status. An example of the former can be found in [16], where the authors study spark-ignition engines in avionics. They develop virtual sensors for quantities for which a physical sensor would have been too expensive to deploy, or too slow at collecting data. They use artificial neural networks to predict measurements from other sensors’ readings. Other authors use virtual sensors to estimate tire forces in automotive systems [17]. They use Kalman filter, ANN and physical relations between measurable quantities in the system such as wheel speed.

An example of virtual sensors used for data validation can be found in [18], where the authors present an approach for sensors data validation and reconstruction and apply it to urban water distribution systems. Raw data undergoes several tests, from low-level tests checking elementary properties of signals to high-level tests exploiting *spatial consistency* between different sensors.

In complex systems, it is not trivial to design effective virtual sensors, due to the large combination of available inputs but also to the diversity of modeling techniques. While a popular approach is to use general purpose simulation software, there is research effort to produce software tools able to create and parametrize modular virtual sensors [19].

Li et al. present a review of virtual sensing techniques in the context of buildings systems [20]. Virtual sensors have been successfully applied to fields such as process control and the automotive sector for more than two decades, and buildings systems could benefit from their application. e.g., many of the FDD techniques proposed for buildings cannot be applied in practice due to sensors not available in real buildings or not accurate enough. Virtual sensors can be used to overcome these difficulties and generate high-quality measurements.

**Figure 3:**
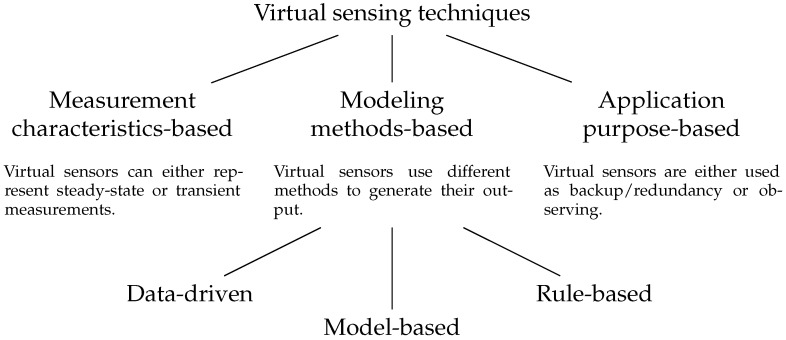
Categorization of virtual sensing techniques according to Li et al. [20].

Virtual sensing techniques are categorized according to three different criteria as shown in Figure 3: measurement characteristics-based, modeling methods-based and application purpose-based. In the measurement characteristics category, virtual sensors can either represent steady-state or transient measurements. In the former case, the model is based on the assumption that the system responds instantaneously to input variables, or that the measured quantities change slowly compared to the system’s dynamics. In the latter case, slower reactions and faster variating input variables are taken into account.

In modeling method category virtual sensors techniques can be divided into model-based and data-driven, similarly to FDD methods. In model-based techniques, detailed knowledge about the system such as mathematical relations between sensors is used to create a model of the sensor. In data-driven techniques, historical data is used to train a black-box model of the system. Methods that are based both on physical models and data trained models are called gray-box models.

With respect to application purposes, virtual sensors are either used as backup/redundancy or observing. In the former case, virtual sensors measure quantities for which other physical sensors exist. They can be used to validate such physical sensors’ readings together with FDD methods or to replace them if they fail. In the latter case, virtual sensors measure quantities unknown or even non-measurable in the system, such as performance or efficiency, and make them available to client applications.

On ventilation units specifically, virtual sensors have been used both to measure unknown quantities and to perform FDD. An example of system monitoring can be found in [21], where the authors develop a virtual sensor modeling exhaust airflow. Airflow sensors for exhaust duct are rarely present in ventilation units due to their cost. They use energy balance equation to relate other sensors in the system with the airflow and propose two different models. While the local errors can be large, the authors show how the cumulative residuals are small and, therefore, the virtual sensor can be used to estimate daily averages.

In [22] the authors report how using virtual sensors significantly improves FDD performance for HVAC systems. They propose a multi-model FDD method that exploits components interdependencies. They develop Bayesian networks for multiple operating modes, using both physical and virtual sensors created from system knowledge and historical data.

Other buildings subsystems have been considered for FDD using virtual sensors. The method proposed in [23] is applied to air conditioners using features decoupling and virtual sensors. The authors create virtual sensors for several quantities, such as compressor power consumption, refrigerant flow, condenser exit pressure, exit air humidity and evaporation temperature. Virtual sensor performances are tested both at steady state and under transients.

A method for FDD on air conditioners is proposed in [24]. The author develops three different virtual sensors for virtual refrigerant charge sensors using different techniques. Information from laboratory tests and manufacturers’ data was used to assess the impact of faults on system performance. A complete implementation was provided for a rooftop air-conditioning unit.

While not part of ventilation units themselves, room-level sensors, i.e., temperature, CO_2_ level and relative humidity, are essential to their correct operation. In [25] a data-driven model for virtual sensors for room-level indoor air conditions is proposed. The authors develop four data mining techniques, including artificial neural network, support vector machine regression and Pace regression. The obtained virtual sensors can be used for validation and calibration of physical sensors.

The reviewed state of the art shows that virtual sensors are popular in the field of buildings systems; however, to our knowledge there is no work so far on employing data-driven virtual sensors for fault detection and diagnostics application on ventilation units. Most of the work reviewed covers other buildings subsystems, such as chillers and air-conditioning units [20,23,24], boilers [22], heat pumps [20] and room-level components [25]. Moreover, in ventilation units, virtual sensors are usually developed to provide readings for unmeasured quantities [21], and when they are considered for explicit application for fault detection and diagnostics they are designed using first principles methods [20]. Other approaches focus on a higher level of diagnostics and require significant expert knowledge to define fault and symptoms [22]. *Therefore, the main contribution of this paper is a specific fault detection and diagnostics application for ventilation units based on virtual sensors created using a data-driven approach*.

## 3. Material and Methods

In this section, we describe the proposed method for FDD on ventilation units based on virtual sensors.

A ventilation unit is an aggregate of several components, integrated together to provide air exchange for the building. It is important that every component works correctly, otherwise the performance of the unit will deteriorate, causing energy loss and reducing comfort level in the building.

Since all components work together, they exhibit common patterns and shared phenomena. Even if there is no explicit redundancy in the system, i.e., no duplicated sensor or meter, many of the quantities in the unit are strongly correlated. In this paper, we propose to exploit these relations and create models to predict a quantity from the surrounding ones, generating a set of *virtual sensors*. Given physical sensors available in the ventilation unit $S_{1},S_{2},\cdots ,S_{n}$, a virtual sensor $S_{i}^{\prime }$ measuring the same quantity of $S_{i}$ is created using a model f(·) that takes other sensors as input, i.e.,

(1) $$\begin{array}{cc} S_{i}^{\prime } & =f(\bar{S})\\ \bar{S} & ⊊\{S_{1},S_{2},\cdots ,S_{i-1},S_{i+1},\cdots ,S_{n}\}. \end{array}$$

For instance, consider a heating system where the following quantities are measured with sensors or meters: initial temperature $T_{0}$, heater energy *M* and final temperature $T_{f}$. A virtual sensor for final temperature could be created using a model of initial temperature and heater energy $T_{f}^{\prime }=f\left(M,T_{0}\right)$. In principle, virtual sensors can be created for any measure inside the system under test, it is not a requirement that a real sensor exists.

Different methods can be used to compute the value of a virtual sensor. When detailed knowledge about the unit is available it is possible to use physical models, e.g., computing airflow using fan speed and duct size and shape. Otherwise, it is possible to train black-box models using data-driven techniques such as regression models, artificial neural network or support vector machine.

**Figure 4:**
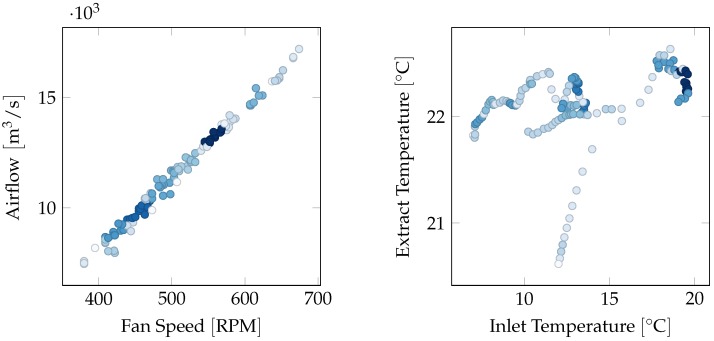
Density plots showing correlations between fan speed and airflow, and between inlet and extract temperatures. Darker colors correspond to more frequent readings. The quantities on the left plot are highly correlated, while the ones on the right one are essentially independent on each other.

A ventilation unit contains several sensors necessary to its functions, such as temperature sensors at various locations, airflow and fan speed at each fan and pump, and energy meters for different components. However, not all of them are closely related to each other and, therefore, it is important to carefully design each virtual sensor by choosing quantities that are correlated. e.g., as shown in Figure 4, fan speed and airflow through the same fan are obviously highly correlated, while inlet air temperature and extract air temperature are independent on each other.

### 3.1. Fault Diagnostics

When two correlated sensors, either physical or virtual, deviate, the only possible inference is that a fault is affecting one of them. To diagnose the faulty one, a third sensor is necessary. Under the assumption of single simultaneous fault, when in a group of three sensors one deviates from the other twos, the former is identified as faulty.

Due to cost and space constraints, duplicated sensors are rarely available in ventilation units, and even less so are triplicated sensors. However, these constraints do not impact virtual sensors, which can be created without cost using data from other components. Some care is necessary when choosing the inputs: different virtual sensors should share as few inputs as possible because a fault in an input impacts all its related virtual sensors.

For instance, consider a heating system with two initial temperature sensors $T_{0},T_{1}$, a heater energy meter *M* and a final temperature sensor $T_{f}$, where two additional virtual sensors for final temperature were created as

(2) $$T_{f}^{\prime }=f\left(M,T_{0}\right),\;T_{f}^{\prime \prime }=f\left(M,T_{1}\right).$$

Assuming a single fault scenario, if $T_{f}^{\prime }$ and $T_{f}^{\prime \prime }$ agree on their readings and $T_{f}$ deviates from them there are two possible causes:

- Sensor $T_{f}$ is faulty;
- Heater energy meter *M* is faulty.

This is because heater energy meter *M* is used as input in both virtual sensors $T_{f}^{\prime }$ and $T_{f}^{\prime \prime }$, therefore, its fault impacts both their output.

### 3.2. Measuring Deviations from Physical Sensors

To automatically detect a fault, a measure of how much the virtual sensors deviate from the physical one is necessary. Several tools are available from statistical analysis, e.g., the maximal error or the norm of residuals. For the first part of the case study, where we use linear regression models to create virtual sensors, we use the coefficient of determination, or $R^{2}$ score, which gives an estimate of how much a linear regression model fits the data [26]. Given a signal $y_{i},i\in \left[1,n\right]$ with mean $\bar{y}$ and its predictions ${\hat{y}}_{i}$ the $R^{2}$ score is defined as

(3) $$\begin{array}{cc} R^{2} & =1-\frac{\mathrm{Sum}\;\mathrm{of}\;{\mathrm{squares}}_{\mathrm{residual}}}{\mathrm{Sum}\;\mathrm{of}\;{\mathrm{squares}}_{\mathrm{total}}}\\ \mathrm{Sum}\;\mathrm{of}\;{\mathrm{squares}}_{\mathrm{residual}} & =\sum_{i=1}^{n}{\left(y_{i}-{\hat{y}}_{i}\right)}^{2}\\ \mathrm{Sum}\;\mathrm{of}\;{\mathrm{squares}}_{\mathrm{total}} & =\sum_{i=1}^{n}{\left(y_{i}-\bar{y}\right)}^{2}. \end{array}$$

An $R^{2}$ score close to 1 indicates that the model is a good fit for the data, while values close to zero indicates the opposite. Negative values indicate that the model predicts data worse than a constant horizontal line.

We use the $R^{2}$ score both to verify that the trained models fit the testing data, i.e., that the designed model accurately follows the physical sensor, and to validate real-time data from the ventilation unit. For each period of interest, e.g., every day, the $R^{2}$ score for each virtual sensor against the physical sensor is recorded. When the measure is lower than a given threshold the pair virtual/physical sensors, are flagged as anomalous or faulty.

**Figure 5:**
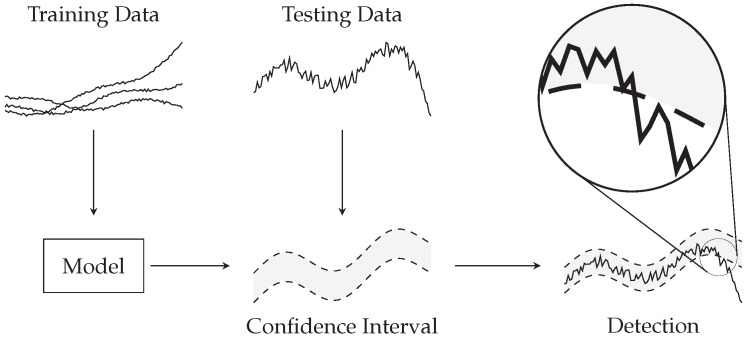
Virtual sensors can generate an expected confidence interval. When readings from the physical sensor fall outside such interval the sensors pair is flagged as anomalous or faulty.

$R^{2}$ score is only meaningful for linear regression models and does not yield useful value for non-linear ones. An alternative option for detecting deviations from the physical sensor is to make the virtual sensor generate an *acceptable range* of values. e.g., the acceptable error could be as large as the largest error obtained when predicting the original training data, or a confidence interval could be built from training data. When readings from the physical sensor fall outside the acceptable range the sensors pair is flagged as anomalous or faulty. This approach is illustrated in Figure 5.

With both approaches, labelled faulty testing data would be necessary to obtain accurate thresholds.

## 4. Results and Discussion

**Figure 6:**
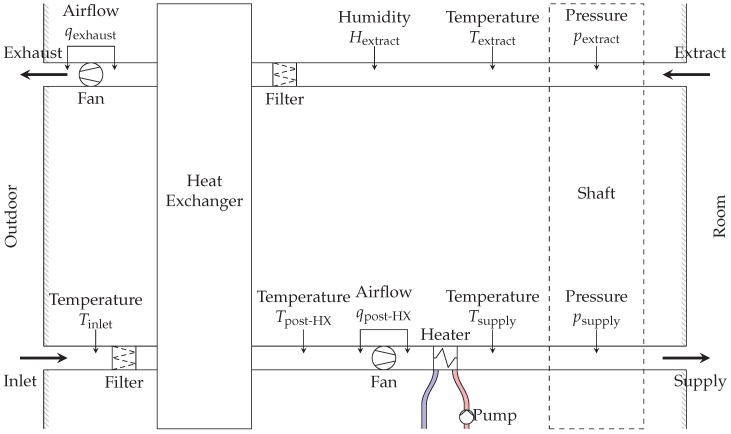
Diagram of a ventilation unit in building OU44. Inlet air enters the unit from bottom-left, passes through the heat-exchanger and through the heater, before entering the main shaft and supplying individual rooms. From the rooms it enters again the main shaft, goes through the heat-exchanger to heat up inlet air, and finally is pushed outside the building. Several sensors, shown by arrows, are available in the unit.

**Figure 7:**
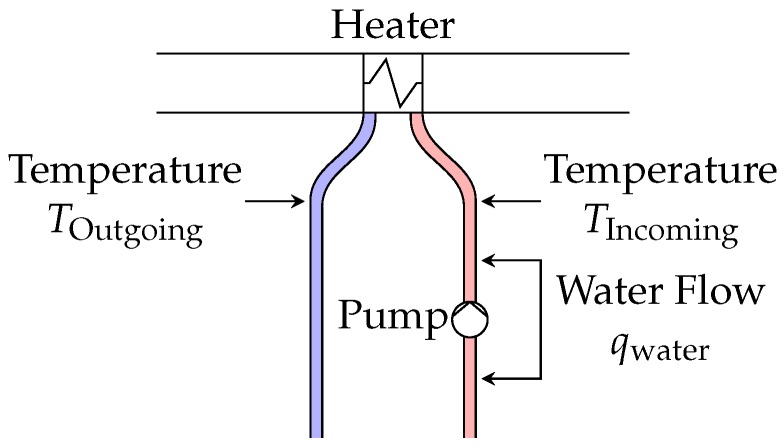
Diagram of a heating loop in building OU44. Hot water is used to heat up the air before it enters the main shaft. Several sensors, shown by arrows, are available in the loop.

In this section, we implement the method presented in Section 3 on a ventilation unit of an existing building. We detail the ventilation unit structure and its sensors and components (Figure 6 and Figure 7). Afterwards, we design three virtual sensors based on linear regression models to duplicate the readings of physical sensors, and we compare physical and virtual readings to detect anomalous behaviors. Finally, we design additional virtual sensors based on statistical and non-linear regression models.

### 4.1. Building OU44

In this paper, we present Odense undervisning building 44 (OU44) as a case study [27]. It was built in 2015 at the University of Southern Denmark, campus Odense, and it is mainly used for teaching. It has three floors plus a basement and it contains classrooms, study zones, offices, and auditoriums. It has four nearly identical ventilation units, each serving one corner of the building, or roughly 20 thermal zones.

A ventilation unit consists of a large air loop, as shown in Figure 6. Inlet air enters the building, goes through a heat-exchanger (HX), then is heated to an appropriate indoor temperature and pushed to the supply shaft, which is connected by variable air volumes (VAVs) units to individual rooms. In the same way, exhaust air is collected from individual rooms in the extract shaft, it goes through the heat-exchanger and it is pushed outside. The heat-exchanger recovers heat from exhaust air and transfers it to inlet air, reducing the energy required by the heater. Air pressures in supply and extract shafts are kept at constant values 130 Pa and 40 Pa, which cause air to flow in the rooms. Two fans in the ventilation unit generate the required airflows to maintain the pressure setpoints.

Heaters, shown in Figure 7, use a hot-water loop, provided by a district-heating system, to heat air inside the ventilation unit.

Several sensors, shown as arrows in Figure 6 and Figure 7 are available inside ventilation units and heating loops: air temperature at several positions, airflows through the two fans, supply and extract pressure, incoming and outgoing water temperature, and water flow through the pump. In addition to that, several meters measure the activity of fans and water pump: fan speed $\omega_{\mathrm{exhaust}/\mathrm{post-HX}}$, fan current $i_{\mathrm{exhaust}/\mathrm{post-HX}}$ and voltage $V_{\mathrm{exhaust}/\mathrm{post-HX}}$, fan power and electrical consumption, and pump electrical consumption.

Ventilation units only function during working hours, i.e., from Monday to Friday from 7 a.m. to 6 p.m. in local time. At night and during weekends they are shut down.

### 4.2. Results Using Linear Regression Models

Three sensors were considered for monitoring in a ventilation unit: post-heat-exchanger temperature, airflow, and fan speed. For each of them, two different models were constructed using other sensors as inputs, as shown in Table 1. Linear regression models were used under the assumption that inputs and outputs obey linear relations, at least locally [28]. Since the periodicity of the system’s behavior is one week, models were trained over a week-long historical data from Monday 13 March 2017 to Sunday 19 March 2017 and tested over two weeks from Monday 27 March 2017 to Sunday 9 April 2017. This period was one of the longest ones with continuously available data for every sensor in each ventilation unit. Training and testing periods were within the same month, therefore, no significant seasonal variation that could influence the models was expected. Additional care should be taken when this assumption does not hold, e.g., in this particular case a teaching building could be configured to operate differently during summer vacations.

For both training and testing phases, raw data from the building management system was resampled to a common, fixed period of 10 min. This step was necessary because the various sensors inside the ventilation unit do not report at the same exact time. Regression models, on the other hand, require readings from different time-series to be simultaneous. No other preprocessing operations were performed. In particular, no faults were artificially added to data.

Another virtual sensor was also constructed, i.e., *Effort* (eff), which is proportional to an estimate of the power requested to the ventilation unit and, therefore, to the airflow. By design, fans produce airflow to maintain constant shaft pressure, which in turn depends on how many VAV units are open in the building. When a VAV unit is open it makes air flowing from the supply shaft through the room to the extract shaft, which results in pressure loss. Fans will then increase their speed to make up for such loss. Effort is an aggregate count of those units, which makes it effectively a virtual sensor for an unknown quantity in the ventilation unit, and is defined as

(4) $$\begin{array}{cc} \mathrm{eff} & =\sum_{i\in \mathrm{VAV}\;\mathrm{units}}\tau_{i}\\ \tau_{i} & =\mathrm{openness}\;\mathrm{ratio}\;\mathrm{of}\;\mathrm{VAV}\;\mathrm{unit}\;i\\ q & \propto \mathrm{eff}\;\Delta p. \end{array}$$

Table 2 shows the coefficients obtained for models with multiple input variables. Most variables have coefficients significantly larger than their standard deviation, therefore, they are significant in their relative models. Two exceptions are water flow and incoming water temperature in Model B, whose contributions are smaller.

**Figure 8:**
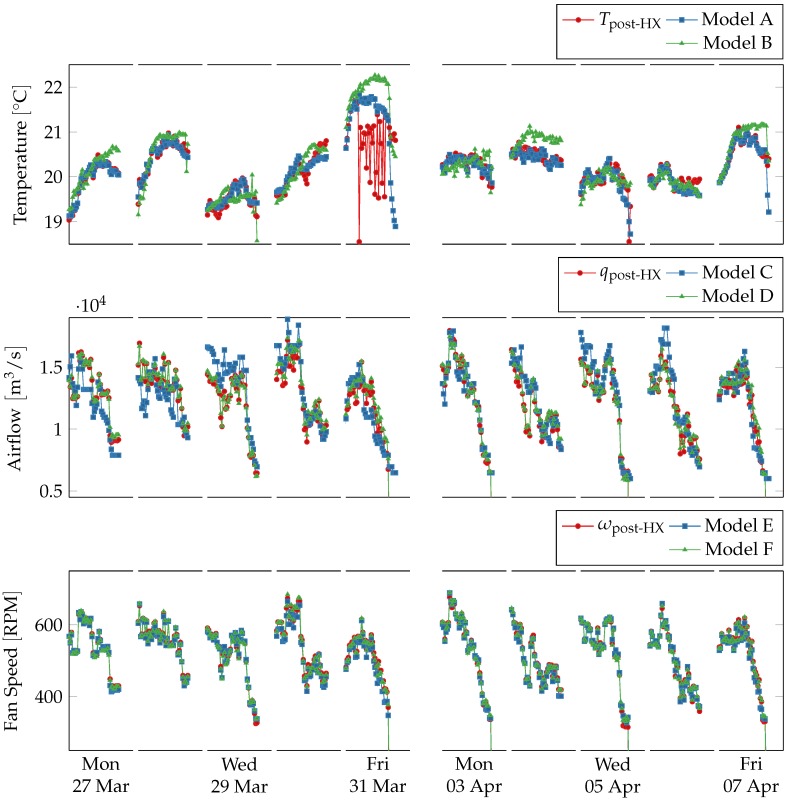
Comparison between physical sensors and linear regression model-based virtual sensors for post heat-exchanger temperature, airflow, and fan speed during working hours (from 8 a.m. to 5 p.m.) over two weeks. Outside working hours and during weekends the ventilation system is shut down. The virtual sensors follow the physical ones except in two cases. On Friday in the first week the temperature sensor oscillates strongly and deviates from the two virtual sensors. On Tuesday in the second week the virtual sensors Model B consistently overestimates the sensors readings.

The three charts in Figure 8 show results for $T_{\mathrm{post-HS}}$, $q_{\mathrm{post-HX}}$ and $\omega_{\mathrm{post-HX}}$ virtual sensors. Data obtained from physical sensors are plotted against data obtained from the two corresponding linear regression virtual sensors defined in Table 1. Deviation from a single virtual sensor is enough to detect a fault but not to isolate and identify the faulty source, therefore, two virtual sensors were used for each physical one. $R^{2}$ scores between physical and predicted readings, which measure how much physical and virtual sensors agree, were computed over daily data as defined in Equation (3) and are shown in Table 3. Low $R^{2}$ scores, indicating that models deviate from the physical sensors, are highlighted in boldface.

**Figure 9:**
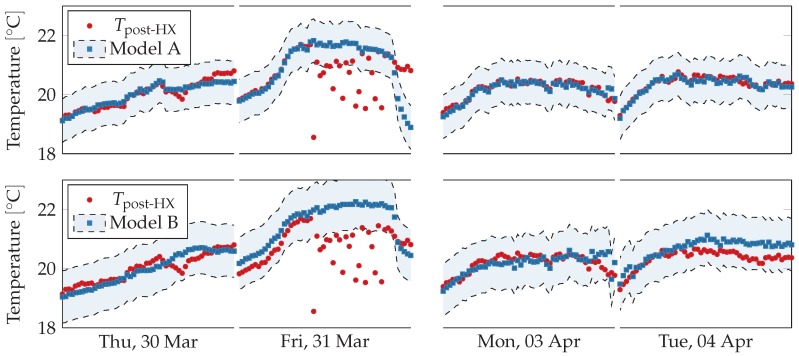
Comparison between physical sensors and acceptable ranges obtained from linear regression model-based virtual sensors for post heat-exchanger temperature during working hours (from 8 a.m. to 5 p.m.) for selected days. The sensors readings fall inside the acceptable ranges except on 31 March 2017, when they deviate significantly. The anomalous trend is not present neither in previous or following days. On 4 April 2017, most models consistently overestimate the physical sensors, but their trends are similar.

For temperature two models are used, one (Model A) exploiting knowledge about the heat-exchanger interactions, using inlet temperature, extract temperature and airflow, i.e.,

(5) $$\begin{array}{cc} \mathrm{Heat} & =c\;\left(T_{\mathrm{post-HX}}-T_{\mathrm{Inlet}}\right)\left(\rho \;\Delta t\;q_{\mathrm{post-HX}}\right)\\ & =c\;\left(T_{\mathrm{Exhaust}}-T_{\mathrm{Extract}}\right)\left(\rho \;\Delta t\;q_{\mathrm{Exhaust}}\right), \end{array}$$

where *c*, ρ and Δt are respectively air specific heat, air density and time step, and other symbols indicate quantities measured by sensors as shown in Figure 6. The other one (Model B) relies on similar but less structured relations between inlet temperature, water flow and temperature difference in the heater. The former predicts temperature value much more accurately than the latter. Table 3 shows that both models deviate significantly from the physical sensor on 31 March 2017, and Model B deviates also on 4 April 2017. Readings from the physical sensors are shown in Figure 9 with respect to the two models’ error ranges, which corresponds to the predictions plus the maximal training error.

**Figure 10:**
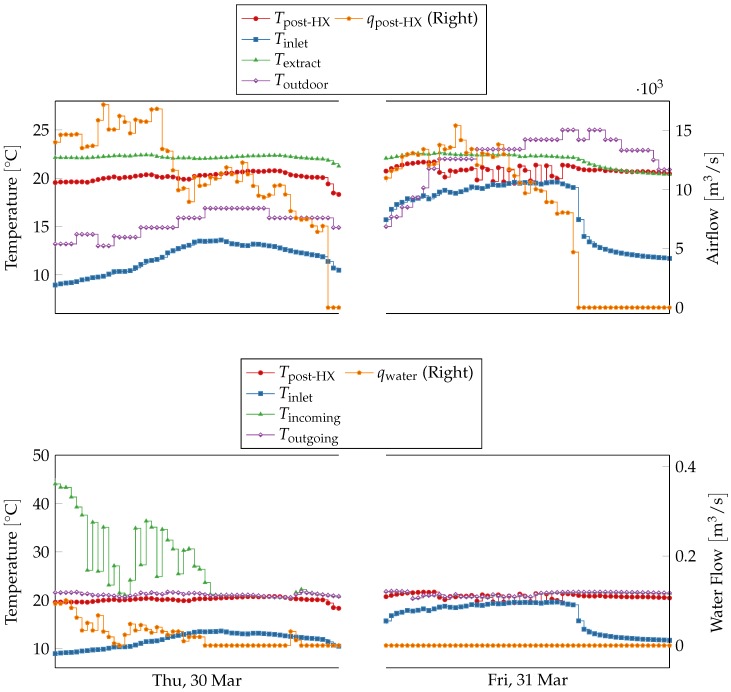
Trends of input and output variables of models A and B on Thursday 30 March 2017 and Friday 31 March 2017. Input variables have similar trends over the two days, but the output variable, post heat-exchanger temperature, exhibits fast oscillation during the second day. Inlet temperature, the shared input variable between the two models, behave similarly over the two days, following the outdoor temperature measured at the local weather station. During the second day the hot water flow is zero, and incoming temperature is equal to outgoing temperature.

On 31 March 2017, the physical sensor’s readings oscillate strongly, in contrast with the two virtual sensors which have a smoother behavior and fall outside the models’ error ranges. Since the two models share an input variable, i.e., inlet temperature, this situation could be caused by a fault in the physical post heat-exchanger temperature sensor or in the inlet temperature sensor. Figure 10 shows the readings for all involved sensors over the faulty period. All measures except post heat-exchanger temperature have smooth trends and behave similarly to the previous day. Inlet temperature rises more than the first day, but it is consistent with outdoor temperature measurements from the local weather station. This suggests that post heat-exchanger temperature is indeed the faulty sensor. The anomalous behavior only lasts for a single day; therefore, this event cannot be classified as a sensor failure, and it could be due to an external disturbance. A further on-site investigation would be necessary to finally identify the precise nature of this event.

The situation on 4 April 2017 is less extreme. Model B consistently overestimate the physical sensor’s readings, but the overall trend is similar and, moreover, all the readings fall inside the model’s error range. Therefore, this event could be classified as a false alarm. Using a more accurate model instead of Model B could reduce the frequency of false alarms.

For airflow two models are used, one using only effort as input (Model C) and one using only fan speed as input (Model D). Airflow and fan speed follow the fan laws and are proportional to each other [29], which could also be inferred from Figure 4, and as expected predictions for this model are nearly exact.

**Figure 11:**
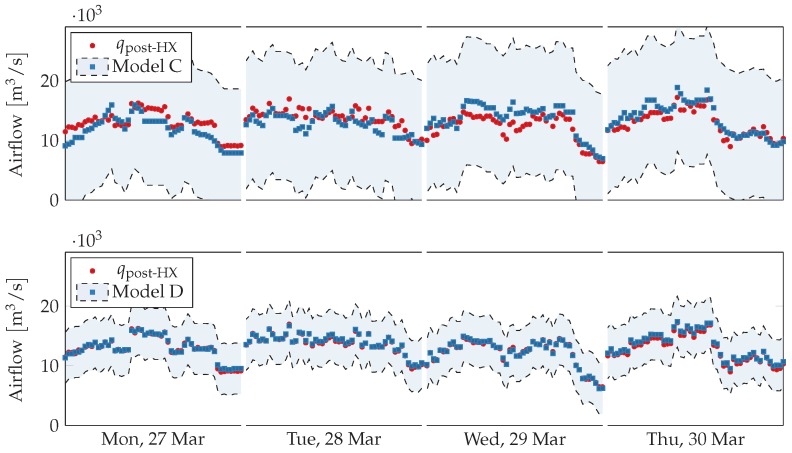
Comparison between physical sensors and acceptable ranges obtained from model-based virtual sensors for post heat-exchanger airflow during working hours (from 8 a.m. to 5 p.m.) for selected days. On Tuesday 28 March 2017 Model C deviates significantly from the physical sensor, but readings always fall inside the acceptable range for the entire period.

Model C is less accurate, and its $R^{2}$ score on Tuesday 28 March 2017 is very low, which suggests a fault in the virtual sensor’s input, i.e., ventilation effort, since Model D agrees with the physical sensor on the same day. Ventilation effort is produced by aggregating several independent streams with frequent periods of missing data, which can indeed cause the model to deviate from the physical sensor. Moreover, ventilation effort does not take into account the size of each room and the corresponding VAV dampers, which reduces the model’s accuracy. Readings from the physical sensors are shown in Figure 11 with respect to the two models’ error ranges, which corresponds to the predictions plus the maximal training error.

For fan speed two models are used, one using airflow as input (Model E) and one using fan current and voltage as inputs (Model F). Fan speed is proportional to airflow due to fan laws, and also proportional to the fan power consumption, which in turn depends on current and voltage W=VI. The former model is nearly exact, for the same reasons explained when discussing Model C. The latter model estimates the power used by the fan, which in turn is correlated with the fan speed, and produces accurate results as well.

### 4.3. Results Using Other Models

While linear regression models were able to detect unusual behavior of post-heat-exchanger temperature sensor, in some cases they did not accurately predict the values of physical sensors. Four additional models were created, as shown in Table 4: two using ARMAX method from statistical analysis [30], and two using non-linear regression methods support vector machine (SVM) [31] and artificial neural network (ANN) [32]. The two approaches augmented linear regression models along two different directions: ARMAX models are linear models over exogenous variables, but they take the endogenous variable’s recent trend into account; ANN and SVM models can instead perform non-linear regression by projecting input data to higher dimensional spaces through non-linear transformations and then performing linear regression. ANN and SVM have both been successfully used in FDD [12,13,14].

#### 4.3.1. ARMAX Models

Models ARMAX A and ARMAX B were trained using post-heat-exchanger temperature as endogenous variable and input sensors from respectively models A and B as exogenous variables. Models SVM and ANN were trained using the same inputs as Model B. As for linear regression models, they were trained over a week-long historical data from Monday 13 March 2017 to Sunday 19 March 2017 and tested over two weeks from Monday 27 March 2017 to Sunday 9 April 2017. As for the experiment with linear regression models, raw data was resampled to a common, fixed period of 10 min.

In ARMAX models data belonging to nights and weekends were removed, i.e., the dataset consisted of continuous working hours. Working and non-working hours correspond to significantly different operation profiles, and since ARMAX methods predict future values based on recent history, they would not perform well when predicting across both. Two different model should instead be created, one for each profile. Since the ventilation system is turned off during non-working hours, in this paper we ignored this case, but in more complex situations where working hours are not fixed, e.g., they depend on the weekday, it would be necessary to split the dataset into distinct parts corresponding to each profile.

**Figure 12:**
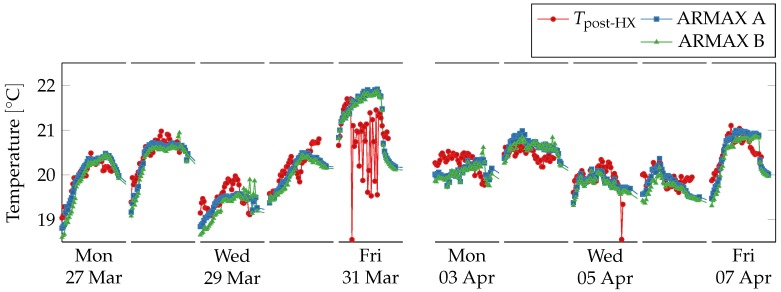
Comparison between physical sensors and ARMAX model-based virtual sensors for post heat-exchanger temperature, airflow, and fan speed during working hours (from 8 a.m. to 5 p.m.) over two weeks. Outside working hours and during weekends the ventilation system is shut down. The virtual sensors follow the physical ones except in one case. On Friday in the first week the temperature sensor oscillates strongly and deviates from the two virtual sensors.

Data sampling period was 10 min, model orders were set to (p,q,d)=(20,2,0) and prediction horizon was set to one working day, i.e., 10 h. Virtual sensors readings are shown against physical sensors readings in Figure 12. The virtual sensors follow closely the physical sensor, except on Friday 31 March 2017 and on Monday 3 April 2017. During the former day, the physical sensor strongly oscillates while the virtual sensors predict a regular trend, in agreement the linear regression virtual sensors. On the latter day, the virtual sensors seem to fail to capture the rising and falling trend from the physical sensor, predicting a straighter line.

**Figure 13:**
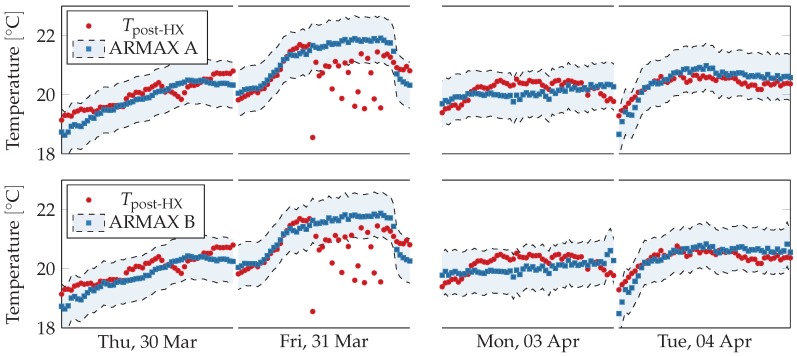
Comparison between physical sensors and acceptable ranges obtained from ARMAX model-based virtual sensors for post heat-exchanger temperature during working hours (from 8 a.m. to 5 p.m.) for selected days. The sensors readings fall inside the acceptable ranges except on Friday 31 March 2017, when they deviate significantly. The anomalous trend is not present neither in previous or following days. On Tuesday 4 April 2017, most models consistently overestimate the physical sensors, but their trends are similar.

Readings from the physical sensor are shown in Figure 13 with respect to the two models’ error ranges, which corresponds to the predictions plus the maximal training error. On Friday 31 March 2017, the physical sensor’s readings fall far outside the acceptable range, which suggests a fault in the sensors pair. On Monday 3 April 2017, despite the trends being different, all readings fall inside the acceptable range.

#### 4.3.2. Non-Linear Regression Models

Model SVM uses support vector machine regression with radial basis function kernels and parameters set to C=100,γ=0.04. Model ANN uses an artificial neural network with 200 hidden layer neurons. Parameters for both models were optimized over the training periods. Only working hours were considered, as with the other models. Both models use the inputs as the Model B described in Table 1.

**Figure 14:**
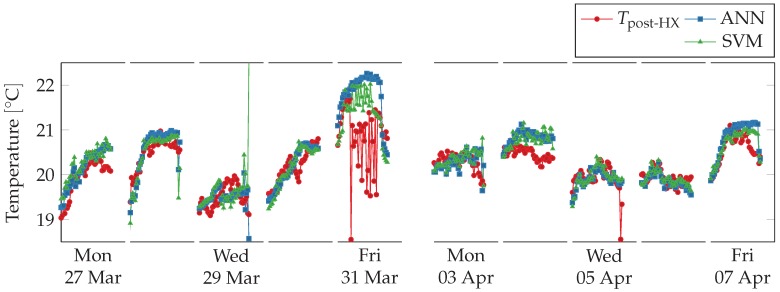
Comparison between physical sensors and non-linear regression model-based virtual sensors for post heat-exchanger temperature, airflow, and fan speed during working hours (from 8 a.m. to 5 p.m.) over two weeks. Outside working hours and during weekends the ventilation system is shut down. The virtual sensors follow the physical ones except in two cases. On Friday in the first week the temperature sensor oscillates strongly and deviates from the two virtual sensors. On Tuesday in the second week the virtual sensors ANN and SVM consistently overestimate the sensors readings.

Virtual sensors readings are shown against physical sensors readings in Figure 14. The virtual sensors follow closely the physical sensor, except on Friday 31 March 2017 and on Tuesday 4 April 2017. During the former, day the physical sensor strongly oscillates while the virtual sensors predict a more regular trend, in agreement the linear regression virtual sensors. Model SVM also predict oscillations, but significantly weaker than the physical sensor. On the latter day, the virtual sensors consistently overestimate the physical one, as it happens with Model B.

**Figure 15:**
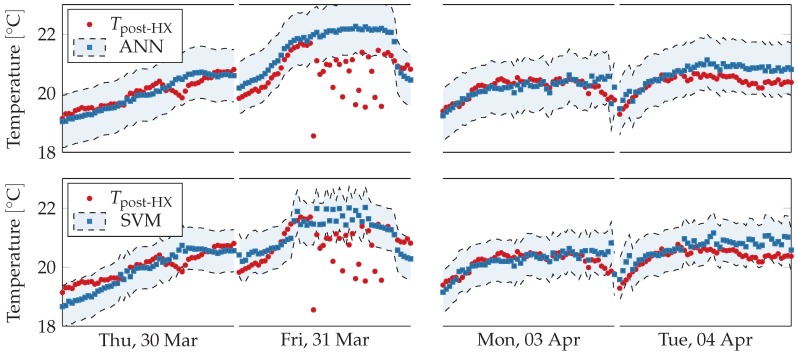
Comparison between physical sensors and acceptable ranges obtained from non-linear regression model-based virtual sensors for post heat-exchanger temperature during working hours (from 8 a.m. to 5 p.m.) for selected days. The sensors readings fall inside the acceptable ranges except on Friday 31 March 2017, when they deviate significantly. The anomalous trend is not present neither in previous or following days. On Tuesday 4 April 2017, most models consistently overestimate the physical sensors, but their trends are similar.

Readings from the physical sensor are shown in Figure 15 with respect to the two models’ error ranges, which corresponds to the predictions plus the maximal training error. On Friday 31 March 2017, the physical sensor’s readings fall far outside the acceptable range, which suggests a fault in the sensors pair. On Monday 3 April 2017, despite virtual sensors overestimate the physical ones, all readings fall inside the acceptable range.

## 5. Conclusions and Future Directions

### 5.1. Conclusions

We proposed a technique to exploit relations between physical quantities inside a ventilation unit to create virtual sensors, introducing, therefore, virtual redundancy. We applied this technique to ventilation units in a real building, creating virtual sensors for each of three existing sensors: temperature, airflow, and fan speed. We applied our method to one of the ventilation units in an existing building and we noted how on a particular day all virtual sensors for temperature, regardless of the model and input sensors used, deviated from the physical sensor. Its trend was, therefore, detected as anomalous.

Virtual sensors can be developed using a multitude of diverse models, with varying accuracy in predicting physical quantities in the system. At first, we employed linear regression models, under the assumption that the related quantities obey linear relations, at least locally. Afterwards, we used ARMAX methods from statistical analysis, where the current value of a sensor was predicted from its history together with the input sensors. Finally, we developed two virtual sensors using non-linear models such as SVM regression and ANN.

We proposed two different techniques to measure deviations between physical and virtual sensors. $R^{2}$ score estimates how good a linear model fits some data. For non-linear models, the $R^{2}$ score is meaningless, therefore, we also used acceptable ranges obtained from the maximal training error. The virtual sensors predicted the values of physical sensors with satisfactory accuracy, and large deviations corresponded to actual anomalous behavior.

Contrary to physical redundancy, virtual redundancy does not increase cost and complexity but carries similar advantages, and several applications can profit from it. e.g., fault detection and diagnostics (FDD) methods, such as the one proposed in this paper, and automatic FDD methods can compare duplicated signals and detect when they diverge from each other. Fault-tolerant control can be achieved by duplicating a physical sensor with a virtual one, so that the system can continue functioning even if it fails. Sensors fusion enhances readings from a physical sensor with readings from other ones, improving measurement accuracy. Expensive physical sensors can be replaced by virtual ones in constrained systems, reducing costs and complexity.

In modern buildings, what sensors should be included in a ventilation unit is currently an open question. Sensors can be expensive and increase the construction complexity of a ventilation unit; however, they are necessary for its correct operation and useful for diagnostics. Virtual sensors are a promising technique that can decrease cost and complexity without compromising functionality or decreasing reliability.

The proposed methodology suffers, however, from some limitations. Data must be available both to create the virtual sensors’ models and to monitor the ventilation unit. Therefore, a system for data collection and storage must be set in place, which could be difficult for older buildings. Data collection should be reliable, i.e., periods of missing data, or ‘data holes’ should be rare, and readings should be validated to ensure the models correctly represent the system. Choosing inputs for virtual sensors model is challenging, and so is choosing the type of model. Complex models can be accurate, but also difficult to develop and can have parameters to estimate, while simple models may not be able to reproduce the entire dynamics of the system.

### 5.2. Future Directions

While the application of the presented method for FDD on ventilation units using virtual sensors yielded promising results, more work is necessary to design and implement an automatic FDD framework. Automatic FDD is necessary to reduce operation cost and increase energy efficiency of buildings [33]. Moreover, comprehensive experiments should be set up to assess the actual benefits of this method [34].

We performed manual FDD by noticing how for one day the $R^{2}$ score between physical and virtual temperature sensors changed abruptly and significantly, and physical sensors’ readings fell outside the acceptable range, which suggested a fault. However, a proper threshold system must be set up to achieve automatic FDD. This can be achieved by using expert knowledge and a training set of labelled faulty historical data, or by generating faulty data using simulations. Moreover, the temperature sensor exhibited faulty behavior only for a single day during the first week, while it appeared to work correctly for the rest of the testing period. Therefore, a threshold system should also be used to decide whether a significant but short-lived deviation is a fault.

We developed virtual sensors using linear and non-linear regression models, together with statistical analysis techniques. Better performance could be achieved by using more advanced methods, such as simulation using energy models of the ventilation units [35]. Moreover, to decide what inputs to use for virtual sensors, we reasoned about the physical relations between quantities inside the ventilation unit. While this approach might lead to accurate results, it could be ineffective for more complex systems. An automatic method could be employed to automatically select inputs and design effective virtual sensors, such as the one presented in [19].

We used regression models to predict data during a period close to the one used for training, under the assumption that the system’s behavior did not change significantly. When extending the prediction to other periods, this assumption might not hold anymore, and seasonal variations must be taken into account.

Finally, in this paper we applied the proposed methodology to sensors in a ventilation unit. Other buildings subsystems could benefit from virtual sensors, e.g., heating loops, lighting, or room-level equipment. Additional work would be necessary to identify inputs and models and to extend the methodology to each of such subsystems.

## Figures and Tables

**Table 1:** Virtual sensors definitions for linear regression models.

Model Name	Output	Inputs
Model A	$T_{\mathrm{post-HX}}$	$T_{\mathrm{inlet}}$, $T_{\mathrm{extract}}$, $q_{\mathrm{post-HX}}$
Model B	$T_{\mathrm{post-HX}}$	$T_{\mathrm{inlet}}$, $q_{\mathrm{water}}$, $T_{\mathrm{incoming}}$, $T_{\mathrm{outgoing}}$
Model C	$q_{\mathrm{post-HX}}$	eff (Equation (4))
Model D	$q_{\mathrm{post-HX}}$	$\omega_{\mathrm{post-HX}}$
Model E	$\omega_{\mathrm{post-HX}}$	$q_{\mathrm{post-HX}}$
Model F	$\omega_{\mathrm{post-HX}}$	$i_{\mathrm{post-HS}}$, $V_{\mathrm{post-HX}}$

**Table 2:** Coefficients for linear regression models.

Variable	Coefficient
**Model A** ($T_{\mathrm{post-HX}}$)
$T_{\mathrm{inlet}}$	0.49 ± 0.012
$T_{\mathrm{extract}}$	0.23 ± 0.017
$q_{\mathrm{post-HX}}$	9.86 × $10^{-2}$ ± 1.404 × $10^{-2}$
**Model B** ($T_{\mathrm{post-HX}}$)
$T_{\mathrm{inlet}}$	0.68 ± 0.021
$T_{\mathrm{incoming}}$	−0.05 ± 0.027
$T_{\mathrm{outgoing}}$	−0.16 ± 0.014
$q_{\mathrm{water}}$	0.03 ± 0.026
**Model C** ($q_{\mathrm{post-HX}}$)
eff	2375 ± 90.2
**Model D** ($q_{\mathrm{post-HX}}$)
$\omega_{\mathrm{post-HX}}$	2766 ± 25.0
**Model E** ($\omega_{\mathrm{post-HX}}$)
$q_{\mathrm{post-HX}}$	85.2 ± 0.770
**Model F** ($\omega_{\mathrm{post-HX}}$)
$i_{\mathrm{post-HX}}$	14.84 ± 1.308
$V_{\mathrm{post-HX}}$	71.58 ± 1.308

**Table 3:** Prediction $R^{2}$ score for virtual sensors. Low scores are highlighted in boldface.

Date	T_post-HX_		q_post-HX_		*ω*_post-HX_	
	Model A	Model B	Model C	Model D	Model E	Model F
2017-03-27	0.955	0.782	0.371	0.987	0.988	0.997
2017-03-28	0.989	0.804	**0.04**	0.98	0.977	0.997
2017-03-29	0.839	0.217	0.368	0.992	0.992	0.995
2017-03-30	0.894	0.729	0.681	0.956	0.956	0.996
2017-03-31	**−1.162**	**−1.995**	0.572	0.852	0.908	0.996
2017-04-03	0.86	0.442	0.87	0.967	0.968	0.997
2017-04-04	0.886	**−0.474**	0.644	0.983	0.984	0.997
2017-04-05	0.774	0.57	0.8	0.944	0.953	0.996
2017-04-06	0.73	0.654	0.622	0.988	0.989	0.997
2017-04-07	0.802	0.537	0.772	0.904	0.932	0.996

**Table 4:** Virtual sensors definitions for other models.

Model Name	Output	Inputs
SVM	$T_{\mathrm{post-HX}}$	$T_{\mathrm{inlet}}$, $q_{\mathrm{water}}$, $T_{\mathrm{incoming}}$, $T_{\mathrm{outgoing}}$
ANN	$T_{\mathrm{post-HX}}$	$T_{\mathrm{inlet}}$, $q_{\mathrm{water}}$, $T_{\mathrm{incoming}}$, $T_{\mathrm{outgoing}}$
ARMAX A	$T_{\mathrm{post-HX}}$	$T_{\mathrm{post-HX}}$, $T_{\mathrm{inlet}}$, $T_{\mathrm{extract}}$, $q_{\mathrm{post-HX}}$
ARMAX B	$T_{\mathrm{post-HX}}$	$T_{\mathrm{post-HX}}$, $T_{\mathrm{inlet}}$, $q_{\mathrm{water}}$, $T_{\mathrm{incoming}}$, $T_{\mathrm{outgoing}}$
